# Non-pharmacological interventions for cognitive function improvement in patients with Alzheimer's disease: a best evidence summary

**DOI:** 10.3389/fneur.2026.1888519

**Published:** 2026-09-25

**Authors:** Nana Wang, Lingmin Dang, Wei Chang, Hong Qi, Jingjing Ban, Huimin Guo, Shan Tang

**Affiliations:** 1 College of Nursing, Shanxi Medical University, Taiyuan, Shanxi, China; 2 General Hospital of Tisco, Sixth Clinical Medical College of Shanxi Medical University, Taiyuan, China; 3 Nursing Department, First Hospital of Shanxi Medical University, Taiyuan, Shanxi, China

**Keywords:** Alzheimer's disease, cognitive function, evidence summary, evidence-based nursing, non-pharmacological interventions

## Abstract

**Objective:**

This study aimed to systematically retrieve, analyze, and synthesize evidence on non-pharmacological interventions to improve overall cognitive function in patients with Alzheimer's disease (AD), providing evidence-based references for selecting and optimizing these interventions.

**Methods:**

This review followed the PRISMA-ScR guidelines and the JBI scoping review framework. The evidence-based question was developed using the PIPOST framework. Databases and professional platforms were systematically searched from inception to January 30, 2026, using the “5S” model. Included studies were quality-appraised; eligible evidence was extracted, synthesized, graded, and assigned recommendation levels.

**Results:**

Following a strict literature screening strategy, a total of 15 studies were included, comprising 1 guideline, 7 expert consensus reports, 6 systematic reviews, and 1 randomized controlled trial. A total of 18 best evidence items were summarized and categorized into six dimensions: cognitive training (CT) and digital therapy, neuromodulation techniques, exercise interventions, lifestyle and reminiscence therapy interventions, music and sensory stimulation, and acupuncture and combined interventions.

**Conclusions:**

This study consolidated the best evidence on non-pharmacological interventions to improve overall cognitive function in patients with Alzheimer's disease, providing references for clinical practice. Clinical healthcare professionals should select appropriate interventions based on the actual clinical scenarios and individual patient conditions, and strengthen quality control during implementation.

## 1 Introduction

Alzheimer's disease (AD) is a neurodegenerative disorder of the central nervous system characterized by a progressive transition from independence to dependence. It is the most common form of dementia among older adults (1, 2). Research indicates that the number of prevalent cases of AD and other dementias in China is approaching 17 million. From 1990 to 2021, the prevalence rate rose from 59.8 per 100,000 to 204.8 per 100,000, a 242.5% increase (3, 4). As the disease progresses, patients may experience memory decline and impaired cognitive function. If left untreated, these symptoms can severely affect physical and mental health and activities of daily living (5, 6). In 2021, the disease resulted in a loss of 10.08 million disability-adjusted life years (DALYs) among older adults in China, making it one of the leading causes of disability and mortality in this population. The disease burden now ranks first globally (7). Improving cognitive function is a critical component of comprehensive AD management and can delay cognitive decline and enhance patients' quality of life (8). Pharmacotherapy remains the primary conventional treatment for AD in current clinical practice. Although it achieves certain therapeutic effects, it is associated with notable adverse reactions (9). Some patients may experience symptoms such as diarrhea, nausea, vomiting, decreased appetite, and dizziness. Among these, diarrhea is more prevalent with donepezil, vomiting with rivastigmine, and decreased appetite with galantamine (10). Therefore, experts in related fields, both domestically and internationally, have shifted the focus of diagnosis and treatment toward non-pharmacological interventions (11). As a multidimensional approach, non-pharmacological interventions are valuable for improving cognitive function, inhibiting early key pathological processes, delaying disease progression, enhancing quality of life, and reducing complications (12). However, at present, the clinical efficacy of non-pharmacological interventions still lacks sufficient, systematic, evidence-based support. This study aims to provide evidence-based guidance for selecting and optimizing non-pharmacological intervention programs for AD and for improving patients' cognitive function by conducting a comprehensive search, evaluation, and synthesis of relevant evidence.

## 2 Materials and methods

This study uses an integrative design that combines a scoping review and an evidence summary, following the JBI methodology framework and reporting it according to the Preferred Reporting Items for Systematic Reviews and Meta-Analyses extension for Scoping Reviews (PRISMA-ScR) guidelines (13, 14) to ensure transparency and rigor in the research process. The study protocol was registered with the Fudan University Evidence-Based Nursing Center (ES20269951, http://ebn.nursing.fudan.edu.cn/myRegisterList).

### 2.1 Formulation of the research question

This study formulated the evidence-based question using the JBI Evidence-Based Practice Center's PIPOST model. *P* (Population) referred to patients diagnosed with Alzheimer's disease (AD). *I* (Intervention) referred to methods for overall cognitive improvement (transcranial direct current or magnetic stimulation, exercise, cognitive interventions, digital therapy, diet, psychosocial interventions, music, reminiscence therapy, Traditional Chinese Medicine therapies, acupuncture, etc.). *P* (Professional) referred to doctors, nurses, rehabilitation therapists, neuropsychologists, and social workers involved in the care and rehabilitation of AD patients. *O* (Outcome) used the Mini-Mental State Examination (MMSE), Montreal Cognitive Assessment (MoCA), and Alzheimer's Disease Assessment Scale-Cognitive Subscale (ADAS-Cog) as indicators. *S* (Setting) included hospitals, community medical institutions, and home-based elderly care. *T* (Type of Evidence) consisted of clinical guidelines, expert consensus, best practices, evidence summaries, meta-analyses, systematic reviews, and randomized controlled trials.

### 2.2 Search strategy

This study was based on the “5S” model proposed by Alper and Xing (15, 16), which encompasses five levels: decision support systems, synopses of evidence, evidence-based guidelines, systematic reviews, and original studies. Following the “top-down” principle, the search was conducted hierarchically, starting at the top level to obtain pre-appraised, high-quality evidence to enhance decision-making efficiency. The search covered the following four categories of authoritative resources: International clinical guidelines and professional society platforms, including BMJ Best Practice, UpToDate, the Guidelines International Network (GIN), the National Institute for Health and Care Excellence (NICE), the Registered Nurses' Association of Ontario (RNAO), Alzheimer's Disease International (ADI), and the Joanna Briggs Institute (JBI) Centre for Evidence-Based Healthcare. International academic literature databases, including PubMed, Embase, CINAHL, Cochrane Library, and Web of Science. Chinese guideline platforms, including Medlive. Chinese academic literature databases, including China National Knowledge Infrastructure (CNKI), Wanfang Data, VIP Database, and Chinese Biomedical Literature Database (CBM). In addition, gray literature was searched via the Cochrane Central Register of Controlled Trials (CENTRAL) and Google Scholar, and a snowball search of the reference lists of included studies was performed. The search period spanned from database inception to January 30, 2026. Search terms were developed jointly by bilingual researchers. The search strategy combined subject headings and free-text terms according to each database's indexing characteristics, balancing precision and comprehensiveness. For databases supporting MeSH, standard subject headings were used for core concepts; for others, adapted subject headings and free-text terms were combined. Boolean operators were used in “Topic,” “Title/Keyword,” or “Intelligent Fields” for Chinese databases. For guideline repositories and association networks such as GIN, NICE, RNAO, and ADI, the core terms “Alzheimer's disease” and “Non-pharmacological intervention” were searched directly in advanced search boxes or topic filters. The detailed construction process, illustrated with PubMed as an example, is presented in Table 1. For the population, the MeSH term “Alzheimer's Disease” (#1) was combined with the free-text term “senile/aged dementia” (#2) using “OR” to form #3. For interventions, various expressions of non-pharmacological interventions were fully considered, integrating MeSH terms [e.g., transcranial stimulation, exercise, cognitive training (CT)] as #4 and free-text terms (e.g., non-pharmaco^*^, digital therapy) as #5, then connected with “OR” to form #6. For document types, publication types (e.g., randomized controlled trial, guideline, systematic review) were combined with the MeSH term “Consensus” as #7, while free-text terms (“best practice”, “evidence summary”, “meta-analysis”) were also used as #8 to supplement retrieval and compensate for potential omissions of non-indexed literature by Publication Type filters; these two sets were connected with “OR” to form #9. Finally, the three sets for population, intervention, and document types were linked with “AND” as #10. Adaptation details for other databases are provided in Supplementary Table S1.

**Table 1:** Search strategy for the PubMed database.

Query	Search term
#1	Search: “Alzheimer's Disease”[Mesh] Sort by: Most Recent
#2	Search: (senile dementia[Title/Abstract]) OR (aged dementia[Title/Abstract])
#3	#1 OR #2
#4	Search: ((((((((“Transcranial Direct Current Stimulation”[Mesh])) OR “Transcranial Magnetic Stimulation”[Mesh]) OR “Exercise”[Mesh]) OR “Cognitive Training”[Mesh]) OR “Diet”[Mesh]) OR “Psychosocial Intervention”[Mesh]) OR “Music Therapy”[Mesh]) OR “Acupuncture Therapy”[Mesh] Sort by: Most Recent
#5	Search: (((((non-pharmaco^*^[Title/Abstract]) OR (non-drug[Title/Abstract])) OR (cognitive intervention[Title/Abstract])) OR (digital therapy[Title/Abstract])) OR (reminiscence therapy[Title/Abstract])) OR (traditional Chinese medicine therapy[Title/Abstract])
#6	#4 OR #5
#7	Search: (((“Randomized Controlled Trial”[Publication Type]) OR “Guideline”[Publication Type]) OR “Consensus”[Mesh]) OR “Systematic Review”[Publication Type] Sort by: Most Recent
#8	Search: ((best practice[Title/Abstract]) OR (evidence summary[Title/Abstract])) OR (Meta[Title/Abstract])
#9	#7 OR #8
#10	#3 AND #6 AND #9

### 2.3 Inclusion and exclusion criteria

The inclusion criteria were: (1) patients diagnosed with AD at the dementia stage, or AD dementia subgroup data extracted from mixed populations; (2) studies meeting the diagnostic criteria of the National Institute of Neurological and Communicative Disorders and Stroke-Alzheimer's Diseases and Related Disorders Association (NINCDS-ADRDA) or the revised the National Institute on Aging-Alzheimer's Association (NIA-AA), preferably supported by biomarker evidence (such as abnormal CSF Aβ_42_ and tau protein, and PET amyloid deposition) (17–19); (3) outcome measures included internationally recognized global cognitive rating scales, such as MMSE, MoCA, and ADAS-Cog, to ensure objectivity, comprehensiveness, and comparability across studies; and (4) the types of evidence included guidelines, consensus statements, best practices, evidence summaries, meta-analyses, systematic reviews, and randomized controlled trials. The exclusion criteria were: (1) studies with serious methodological flaws; (2) incomplete information (such as the first author, publication year, publication details, or references) or duplicate publications; (3) outdated versions, guideline interpretations, or direct translations; (4) non-English or non-Chinese language; and (5) studies in which the full text, interventions, or outcome measures were inaccessible.

### 2.4 Quality appraisal of evidence

Two researchers independently appraised the quality of the included studies using appropriate tools tailored to study design, cross-checked the results, and resolved discrepancies through discussion or consultation with a third researcher. Guidelines were evaluated using the Appraisal of Guidelines for Research and Evaluation II (AGREE II) instrument (20), which comprises 23 items across 6 domains rated on a 1–7 scale. Guidelines were graded as A (recommended) if all 6 domains scored ≥60%; B (recommended after modification) if 3 or more domains had a standardized percentage between 30% and 60%; and C (not recommended) if 3 or more domains scored < 30%. Inter-rater reliability was assessed using the intraclass correlation coefficient (ICC). Expert consensuses were appraised using the corresponding tool from the JBI Evidence-Based Healthcare Center (21). The quality of systematic reviews was assessed using A Measurement Tool to Assess Systematic Reviews 2 (AMSTAR-2) (22), which consists of 16 items, with items 2, 4, 7, 9, 11, 13, and 15 considered critical. Confidence in the results was categorized as high, moderate, low, or critically low. The quality of RCTs was evaluated using the Cochrane Handbook (23), which includes 7 items and assesses the risk of bias as “Yes” (low risk), “No” (high risk), or “Unclear” (uncertain).

### 2.5 Data extraction

In accordance with the PRISMA-ScR guidelines (item 11) (14), a structured data extraction form was used to standardize data extraction and recording from the included literature. The extracted data included: (1) basic characteristics of the literature (first author or institution, publication year, source of evidence); (2) type of evidence (guidelines, RCTs, systematic reviews, expert consensuses, etc.); (3) study population and clinical conditions; (4) type, characteristics, and implementation details of interventions; (5) settings (hospitals, communities, homes). During extraction, all inferences and simplified data forms derived from the original literature were explicitly documented. This structured and transparent process ensured data consistency and integrity, providing a reliable foundation for subsequent evidence synthesis and grading.

### 2.6 Evidence synthesis and grading

After data extraction was completed, the first and second authors cross-checked the data and logically organized and integrated the evidence. For multi-source evidence, the synthesis rules (24) were as follows: If conclusions were identical, the most professional and concise opinion was adopted; if content overlapped or was complementary, it was integrated into a unified description for clinical practice without deviating from the original meaning (multiple systematic reviews were qualitatively synthesized only, without pooling effect sizes or grading evidence levels based on the number of reviews); if conclusions were contradictory or inconsistent, levels were assigned using the preset appraisal tools, prioritizing the one with the highest methodological quality, followed by the latest publications in authoritative journals. Finally, the evidence was classified into levels 1–5 according to the JBI 2014 pre-grading system (25), based on the study design of the included literature, with level 1 the highest and level 5 the lowest. The specific study designs for each level were as follows: Level 1 included systematic reviews/meta-analyses of multiple RCTs or experimental studies, or high-quality individual RCTs; Level 2 included systematic reviews of multiple quasi-experimental studies, or high-quality individual quasi-experimental studies; Level 3 included systematic reviews of multiple analytical observational studies, or high-quality individual cohort or case-control studies; Level 4 included systematic reviews of multiple descriptive observational studies, or individual cross-sectional studies, case series, or case reports; and Level 5 included expert opinions, expert consensuses, basic research reports, or inferences based on physiological or laboratory studies.

### 2.7 Formulation of recommendations

The strength of the recommendation was not determined solely by the level of evidence; instead, it was evaluated using the JBI FAME structure (25) across four dimensions: Feasibility (*F*), Appropriateness (*A*), Meaningfulness (*M*), and Effectiveness (*E*), based on evidence quality appraisal and synthesis. By comprehensively considering benefits vs. risks, resource costs, patient preferences, and the local clinical context, the final Grade A or Grade B recommendation was determined. An expert argumentation meeting was held in March 2026 and attended by 7 members: 5 specialist research core members focused on neurorehabilitation and psychological support who underwent evidence-based training; 1 frontline clinical nurse with over 10 years of experience; and 1 associate chief nurse with a neurology background and experience in both nursing management and evidence-based research. The research team convened the experts to discuss evidence quality, target population matching, implementation basis, nursing resource requirements, and patient acceptance. Experts independently scored each piece of evidence on a 1–5 scale. The consistency of expert ratings was tested using Kendall's coefficient of concordance (*W*), and the pass rate for each item (number of agreements/total number of experts × 100%) was calculated as a quantitative supplement. For evidence with disagreements or resource constraints, corresponding adjustments were made based on hospital staffing, information technology infrastructure, patient digital skills, and workflows of relevant departments, with the scope of application noted; for cases where consensus was not reached after discussion, the panel chair made the final decision by integrating the level of evidence with clinical needs. Ultimately, the strength of recommendation for each item was clarified as either Grade A (strong recommendation) or Grade B (weak recommendation) (25). The specific evaluation criteria for FAME dimensions and recommendation strength are presented in Supplementary Tables S2, S3.

## 3 Results

### 3.1 Search results and basic characteristics of included literature

A preliminary search identified 2,235 records. Following the PRISMA-ScR guidelines (14), two researchers independently screened the literature. After the initial screening of titles and abstracts, 1,504 articles were included, and 766 articles were included after full-text rescreening. The inter-rater agreement Kappa values for the two screening stages were 0.991 and 0.915, respectively (both >0.80), indicating good consistency. Disagreements were resolved through discussion to reach consensus. After manually supplementing 3 additional articles, a total of 15 articles were finally included. The literature screening process is shown in Figure 1. The basic characteristics of the included literature are presented in Table 2. (Note: Literature on the ADI platform focusing on non-research targets, such as the living environment of AD patients, has been excluded.)

**Figure 1:**
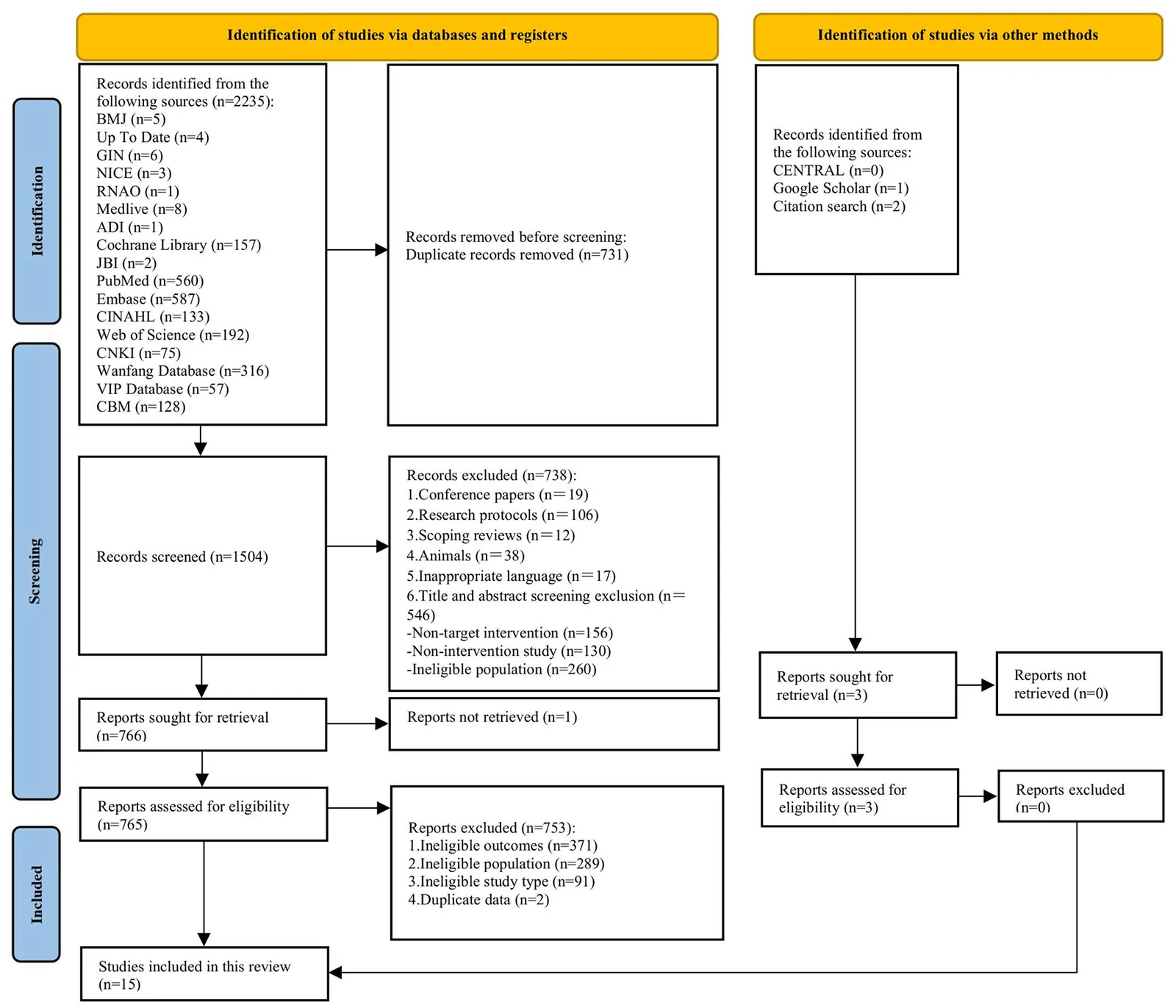
Flowchart of the literature screening process.

**Table 2:** General information of the 15 included articles.

Reference	Year of publication	Evidence source	Type of evidence	Topic of study
Japanese Society of Neurology (26)	2017	UpToDate	Guideline	Clinical practice for dementia (English version)
Academy of Cognitive Disorder of China (27)	2020	Wanfang	Expert consensus	Activity behavior and cognition comprehensive management of Alzheimer's disease
Chinese Expert Consensus on Non-Invasive Neuromodulation Techniques for the Treatment of Alzheimer's Disease Writing Group (28)	2023	CNKI	Expert consensus	Non-invasive neuromodulation techniques for the treatment of Alzheimer's disease
Gong et al. (29)	2025	CNKI	Expert consensus	Non-pharmacological interventions for cognitive impairment
Wang et al. (30)	2025	CNKI	Expert consensus	Diversified rehabilitation interventions for Alzheimer's disease
Alzheimer's Disease Committee of Chinese Aging Well Association, Encephalopathy Drug Research Committee of China Association of Traditional Chinese Medicine (31)	2024	Wanfang	Expert consensus	Integrated traditional Chinese and Western medicine diagnosis and treatment of Alzheimer's disease
Congress of Neuro-Degenerative Diseases Committee of the Chinese Society of Microcirculation, Neuropsychology and Behavioral Medicine Section of Neurology Credit Association of Chinese Medical Association, Neurological Rehabilitation Section of Neurological Credit Association of Chinese Medical Association (32)	2020	Wanfang	Expert consensus	Rehabilitation management of Alzheimer's disease
Chinese Society of Dementia and Cognitive Impairment, Consensus Panel on Digital Therapeutics for Cognitive Impairment (33)	2023	Wanfang	Expert consensus	Digital therapeutics for cognitive impairment
Bawa et al. (34)	2021	PubMed	Systematic review	Efficacy of non-invasive brain stimulation on global cognition in Alzheimer's disease and mild cognitive impairment
Xue et al. (35)	2024	PubMed	Systematic review	Repetitive transcranial magnetic stimulation for Alzheimer's disease
Li et al. (36)	2024	PubMed	Systematic review	Unlocking the potential of repetitive transcranial magnetic stimulation in Alzheimer's disease
Guo et al. (37)	2024	PubMed	Systematic review	Efficacy and safety of acupuncture on cognitive impairment in Alzheimer's disease
Xiu et al. (38)	2024	PubMed	Systematic review	High-frequency repetitive transcranial magnetic stimulation (HF-rTMS) on global cognitive function of elderly in mild to moderate Alzheimer's disease
Zhang et al. (39)	2024	PubMed	Systematic review	Safety and effectiveness of 40 Hz γ-tACS in Alzheimer's disease
Yang et al. (40)	2023	CNKI	Randomized controlled trial	Effect of abdominal acupuncture on cognitive function and serum 5-HT, BDNF, IGF-1 in patients with mild to moderate Alzheimer's disease

### 3.2 Quality assessment of included literature

One practice guideline (26) used a two-way mixed-effects model to calculate the ICC with absolute agreement for average measures. The ICC for the two researchers was 0.920 (95% *CI* 0.813–0.966). Because this exceeds 0.750, the overall quality is considered high. The quality assessment results are presented in Table 3. Among the 7 expert consensus papers (27–33), all items in 1 paper (32) were rated as “Yes,” whereas for the remaining 6 papers (27–31, 33), item 6 was rated as “Unclear” and all other items were rated as “Yes.” The quality assessment results are presented in Table 4. Among the 6 included systematic reviews (34–39), there were significant variations in quality. Specifically, 50.00% did not establish a prior research protocol and revision details before conducting the systematic review (34, 37, 38), and did not evaluate the impact of the risk of bias in individual studies on the results when performing the meta-analysis (34, 35, 37); 33.33% did not adequately explain the heterogeneity discussed (35, 37); and 16.67% had omissions in considering the risk of bias in individual studies (34), providing a list of excluded studies and reasons for exclusion (37), describing the basic information of the included studies in detail (37), and disclosing conflicts of interest (34). The quality assessment results are presented in Table 5. For the 1 included RCT (40), due to the implementation characteristics of non-pharmacological interventions, it was difficult to blind participants and personnel; thus, it was assessed as having a high risk of bias for this item. Meanwhile, the details of allocation concealment were insufficiently described, resulting in an unclear risk of bias, while the risk of bias for the remaining items was low. The results of the quality assessment are shown in Table 6.

**Table 3:** Results of guideline quality assessment (*n* = 1).

Included studies	Standardized percentage of each domain						≥60% (count) number of domains	≤ 30% (count) number of domains	Quality grade
	Scope and purpose (%)	Stakeholder involvement (%)	Rigor of development (%)	Clarity of presentation (%)	Applicability (%)	Editorial Independence (%)			
Japanese Society of Neurology (26)	100	100	97.92	100	93.75	100	6	0	A

**Table 4:** Results of expert consensus quality assessment (*n* = 7).

Reference	Year of publication	1	2	3	4	5	6
Academy of Cognitive Disorder of China (27)	2020	Yes	Yes	Yes	Yes	Yes	Unclear
Chinese Expert Consensus on Non-Invasive Neuromodulation Techniques for the Treatment of Alzheimer's Disease Writing Group (28)	2023	Yes	Yes	Yes	Yes	Yes	Unclear
Gong et al. (29)	2025	Yes	Yes	Yes	Yes	Yes	Unclear
Wang et al. (30)	2025	Yes	Yes	Yes	Yes	Yes	Unclear
Alzheimer's Disease Committee of Chinese Aging Well Association, Encephalopathy Drug Research Committee of China Association of Traditional Chinese Medicine (31)	2024	Yes	Yes	Yes	Yes	Yes	Unclear
Congress of Neuro-Degenerative Diseases Committee of the Chinese Society of Microcirculation, Neuropsychology and Behavioral Medicine Section of Neurology Credit Association of Chinese Medical Association, Neurological Rehabilitation Section of Neurological Credit Association of Chinese Medical Association (32)	2020	Yes	Yes	Yes	Yes	Yes	Yes
Chinese Society of Dementia and Cognitive Impairment, Consensus Panel on Digital Therapeutics for Cognitive Impairment (33)	2023	Yes	Yes	Yes	Yes	Yes	Unclear

1. Was the source of the viewpoint clearly indicated? 2. Did the viewpoint come from influential experts in the field? 3. Was the viewpoint centered on the interests of the relevant population? 4. Were the conclusions based on analytical results, and was the expression of the viewpoint logical? 5. Were references to existing literature taken into account? 6. Did the viewpoint present any inconsistencies with previous literature?

**Table 5:** Results of systematic reviews quality assessment (*n* = 6).

Reference	Year of publication	1	2	3	4	5	6	7	8	9	10	11	12	13	14	15	16	Overall quality
Bawa et al. (34)	2021	Yes	Partial	Yes	Yes	Yes	Yes	Yes	Yes	Yes	Yes	Yes	No	No	Yes	Yes	No	Very low
Xue et al. (35)	2024	Yes	Yes	Yes	Yes	Yes	Yes	Yes	Yes	Yes	Yes	Yes	No	Yes	No	Yes	Yes	Medium
Li et al. (36)	2024	Yes	Yes	Yes	Yes	Yes	Yes	Yes	Yes	Yes	Yes	Yes	Yes	Yes	Yes	Yes	Yes	High
Guo et al. (37)	2024	Yes	Partial	Yes	Yes	Yes	Yes	No	Partial	Yes	Yes	Yes	No	Yes	No	Yes	Yes	Very low
Xiu et al. (38)	2024	Yes	No	Yes	Yes	Yes	Yes	Yes	Yes	Yes	Yes	Yes	Yes	Yes	Yes	Yes	Yes	Low
Zhang et al. (39)	2024	Yes	Yes	Yes	Yes	Yes	Yes	Yes	Yes	Yes	Yes	Yes	Yes	Yes	Yes	Yes	Yes	High

1. Whether the research question and inclusion criteria of the systematic review are based on PICO; 2. whether a preliminary research protocol was developed before conducting the systematic review, and if revised, whether details of the revision are reported; 3. whether the rationale for the choice of study design is explained; 4. whether a comprehensive search strategy was used; 5. Whether study selection is reproducible; 6. whether data extraction is reproducible; 7. whether a list of excluded studies and reasons for exclusion is provided; 8. whether detailed basic information on included studies is described; 9. whether the method for assessing risk of bias in included studies is reasonable; 10. whether funding sources for included studies are reported; 11. if a meta-analysis was performed, whether the statistical methods for result synthesis are appropriate; 12. if a meta-analysis was performed, whether the impact of single-study bias risk on meta-analysis results was evaluated; 13. whether single-study bias risk was considered when interpreting and discussing the results; 14. whether heterogeneity is satisfactorily explained and discussed; 15. if quantitative synthesis was performed, whether the possibility of publication bias was adequately investigated and its impact on results discussed; 16. whether potential sources of conflicts of interest are reported, including funding received for the systematic review.

**Table 6:** Results of randomized controlled studies quality assessment (*n* = 1).

Reference	Year of publication	1	2	3	4	5	6	7
Yang et al. (40)	2023	Low risk of bias	Unclear	High risk of bias	Low risk of bias	Low risk of bias	Low risk of bias	Low risk of bias

1. Random sequence generation; 2. allocation concealment; 3. blinding of participants and researchers; 4. blinding of outcome assessment; 5. completeness of outcome data; 6. selective reporting of results; 7. other biases.

### 3.3 Clinical evaluation and recommendation results

This study ultimately included 15 documents, comprising 7 Level 1 evidence items and 11 Level 5 evidence items. After quality assessment and evidence synthesis, a total of 18 evidence items were extracted. Based on the FAME framework and deliberation by the expert group, 10 items were identified as Grade A recommendations and 8 as Grade B recommendations. Kendall's coefficient of concordance was 0.562 (*P* < 0.001), indicating basic consensus among the evaluators. Evidence items 2 and 7 had an inclusion pass rate of 85.71%, while the rest were 100%. Evidence 2 reached only Level 5 but has the potential to support a full-scenario cognitive intervention system and is an important supplement to non-pharmacological interventions for AD; the expert group decided to retain it and tentatively list it as Grade B, pending verification of efficacy by high-quality studies for future upgrading. Evidence 7 faces limited availability of specialized equipment in China and a lack of configuration at the grassroots level, and requires systematic training for patients and families before home use, limiting its suitability; however, as it is Level 1 with high overall quality and can provide a new pathway for non-invasive intervention in home settings, it was listed as Grade B. It can be expanded upon the maturation of equipment conditions and improvement of the training system; thus, both were included. Evidence 5 is Level 1; although it included a small amount of low-quality literature, this served only as auxiliary reference, with key outcome indicators supported by high-quality literature, consistent conclusions, and high FAME results, so the expert group ruled to maintain it as Grade A. The study designs for Evidence 8 and Evidence 18 were both Level 1 with high FAME ratings, but the former's supporting systematic review had methodological flaws assessed by AMSTAR-2, and the latter originated from a single-center small-sample study and failed to blind participants and researchers during implementation, posing a high risk of bias; both were downgraded to Grade B after comprehensive judgment by the expert group. The remaining items (such as Evidence 6, 12, 14, and 16) were also listed as Grade B due to differences in implementation conditions, patient acceptance, cost-effectiveness, or long-term adherence; the remaining evidence was listed as Grade A strong recommendations.

### 3.4 Final evidence description and synthesis

This study synthesized 18 pieces of best evidence across 6 domains: cognitive training and digital therapy, neuromodulation techniques, exercise interventions, lifestyle and reminiscence interventions, music and sensory stimulation, and acupuncture and comprehensive interventions. See Table 7. The specific JBI FAME evaluation results are shown in Supplementary Table S4.

**Table 7:** Summary of best evidence on the impact of non-pharmacological interventions on cognitive function in patients with Alzheimer's disease.

Evidence dimension	Evidence subject/description	Evidence level/source	Recommendation level/decision
Cognitive training AND digital therapy	1. Cognitive training (CT): using tools such as paper and cards for single-cognitive-domain (attention, memory) training; effects are transferable to other cognitive domains (32)	5 (expert consensus)	A (adopt)
	2. Cognitive digital therapy: prioritize adaptive computerized cognitive training across multiple cognitive domains; can be used alone or combined with virtual reality and physical stimulation to improve overall cognition (33)	5 (expert consensus)	B (revise)
	3. Computer-assisted cognitive rehabilitation (CACR): conduct remote, personalized cognitive training (e.g., memory and attention training) using devices such as computers and mobile phones; can be combined with acupuncture to enhance intervention effects (32)	5 (expert consensus)	A (adopt)
	4. Cognitive stimulation (CST): conduct engaging activities like discussions and word associations in groups to activate cognitive functions (32)	5 (expert consensus)	A (Adopt)
Neuromodulation techniques	5. rTMS: for patients aged ≥60 with mild-to-moderate AD, target the left or bilateral DLPFC, with the parietal lobe as supplement, using high-frequency stimulation at 5, 10, or 20 Hz (28, 29, 31, 35, 36, 38)	1 (systematic review/expert consensus)	A (adopt)
	6. tDCS: anode preferentially targets the left DLPFC (cathode placed on the right); not recommended for solitary use in AD patients (29, 31, 34)	1 (systematic review/expert consensus)	B (adopt)
	7. γ-tACS (40 Hz): 1–1.5 mA excitatory intervention, can be used for home intervention (30, 39)	1 (systematic review/expert consensus)	B (revise)
	8. Comprehensive evaluation: rTMS, tDCS, and γ-tACS (40 Hz) are all effective, with rTMS the preferred non-invasive option for the dementia stage of AD (28, 34)	1 (systematic review/expert consensus)	B (adopt)
Exercise intervention	9. Moderate-intensity aerobic exercise: recommendations include jogging, brisk walking, cycling, swimming, and ball games. Patients with balance impairments require supervision (27, 30)	5 (expert consensus)	A (adopt)
	10. Moderate-intensity strength training: recommended for mild-to-moderate AD patients, using equipment or body-weight resistance training, Tai Chi, and Yoga (26, 30)	1 (expert consensus/guideline recommendation)	A (adopt)
	11. Exercise prescription: mild-to-moderate AD patients are advised to continue regular aerobic exercise for 6 months (31)	5 (expert consensus)	A (adopt)
Lifestyle and reminiscence intervention	12. Lifestyle intervention: on the basis of regular exercise, participate in computer courses, cognitive challenges, and social interactions; moderately restrict calorie intake; follow a Mediterranean diet; reduce intake of high-sugar, high-saturated-fat foods and excessive alcohol (27)	5 (expert consensus)	B (adopt)
	13. Reminiscence and memory guidance: guided by family, friends, and caregivers to recall past events, feelings, and thoughts, activating remote memory and promoting thinking and emotional expression (30)	5 (expert consensus)	A (adopt)
Music and sensory stimulation	14. Dance therapy: adjust forms based on the patient's cognitive and physical status (mild: choreographed or familiar dances; moderate: impromptu or simple rhythm dances; Severe: mainly soothing, passive limb movements or body swaying) (30)	5 (expert consensus)	B (adopt)
	15. Horticultural therapy: patients with mild-to-moderate AD can participate in planting and gardening crafts; those with severe AD should focus on sensory experiences (garden visits, plant appreciation, smelling flowers, touching plants) (30)	5 (expert consensus)	A (adopt)
Acupuncture and comprehensive intervention	16. Simple acupuncture: efficacy is based on Deqi (arrival of Qi); can be combined with Chinese and Western medical treatments (37)	1 (systematic review)	B (adopt)
	17. Combined Intervention: music therapy (e.g., group singing, playing instruments), reminiscence therapy (e.g., using old photos and hometown customs), and neuroregulation; the curative effect is superior to single interventions (32)	5 (expert consensus)	A (adopt)
	18. Combined scalp and abdominal acupuncture: mild-to- moderate AD patients receive basic treatment combined with specific acupoint acupuncture. Scalp acupuncture selects Baihui, Shenting, and Benshen; abdominal acupuncture selects points for “guiding Qi to the source” (Zhongwan, Xiawan, Guanyuan, Qihai), “Four Gates of the Abdomen” (bilateral Huaroumen, bilateral Wailing), and bilateral Tianshu and bilateral Daheng (40)	1 (RCT)	B (adopt)

Explanation of abbreviations and intervention implementation.

1. AD, Alzheimer's disease; CT, cognitive training (CT); CST, cognitive stimulation therapy (CST); CACR, computer-assisted cognitive rehabilitation (CACR) technology; rTMS, repetitive transcranial magnetic stimulation; tDCS, transcranial direct current stimulation; tACS, transcranial alternating current stimulation; DLPFC, dorsolateral prefrontal cortex; RMT, resting motor threshold; MET, metabolic equivalent.

2. Core recommended parameters: (1) rTMS: 80%−120% RMT intensity, 20 min/session, stimulation 1–2 s, interval 20–30 s, single pulse < 2,500, total pulses >20,000, course >20 sessions; special targets can use 40 Hz; 2–12 weeks (MMSE improvement is best at 12 weeks); (2) tDCS: 1–2 mA, 20–30 min/session, 2–6 weeks; (3) Moderate-intensity aerobic exercise: Heart rate 60%−75%, 30–60 min/session, 3–5 times/week; (4) Reminiscence intervention: 1–2 times/week, 30–60 min/session, 8–12 weeks; (5) Dance therapy: low-moderate intensity (< 6 METs), 1–2 times/week, 60 min/session, 3–6 months (can be extended to 12 months); (6) Horticultural therapy: 1–2 times/week, 1–2 h/session, short-term 6 weeks-3 months, long-term 6 months-1 year; (7) Acupuncture: Needle retention 30 min, 8–12 weeks; (8) Combined scalp and abdominal acupuncture: Needle retention of 30 min, with needle manipulation once every 5 min, for 2 months. Scalp acupuncture uses 0.30 mm × 25 mm oblique insertion to 0.5 cun; abdominal acupuncture uses 0.30 mm × 40 mm perpendicular insertion to 1.0 cun.

3. Target population: (1) mild-to-moderate AD patients can use cognitive training, aerobic exercise, reminiscence intervention, rTMS, and combined acupuncture protocols; (2) music, sensory stimulation, and horticultural therapy are suitable for AD patients at all stages; (3) severe cases focus on passive sensory experiences and simplified activities.

4. Implementation settings: (1) rTMS, tDCS, and acupuncture must be administered by medical staff in hospitals or professional institutions; (2) cognitive training (digital therapy), γ-tACS (non-invasive technology), reminiscence intervention, and music therapy can be carried out at home under guidance; (3) cognitive stimulation and exercise are suitable for community implementation.

5. Effect sizes and safety considerations: given the significant clinical heterogeneity across the included studies in stimulation parameters (frequency, intensity), exercise regimens, and control settings, a quantitative meta-analysis to pool effect sizes (with 95% CIs) was not conducted for this evidence summary. Overall, the safety profile is favorable, with adverse events rare and mild. However, attention should be paid to the following: neuromodulation interventions (rTMS, tACS) may cause mild headaches; cognitive training, reminiscence therapy, music, and sensory stimulation may increase the risk of irritability; and exercise interventions require precautions to avoid excessive fatigue or falls.

6. Tips: (1) tDCS is not recommended for use alone in AD patients; (2) home neuromodulation equipment requires professional training and safety assessment; and (3) grassroots translation requires verification of scene adaptability.

## 4 Discussion

This study defined the level of evidence and the strength of recommendation separately: the former refers to the level of research design in the literature, while the latter is a comprehensive judgment based on the quality of the evidence, benefits and risks, resource utilization, patient preferences, and clinical significance. Accordingly, some high-level evidence may be classified as Grade B if it is difficult to implement because of resource constraints or patient acceptance, while individual low-level evidence can receive a stronger recommendation under certain conditions if it can be implemented smoothly, involves little risk, and has clear clinical value. At the same time, countermeasures proposed to address practical obstacles in clinical translation provide a reference for narrowing the gap between evidence and practice.

### 4.1 Cognitive-targeted interventions are the cornerstone for improving cognitive function in patients with AD

(1) Cognitive training and digital therapeutics are foundational intervention methods for improving cognitive function in AD patients: Evidence 1–4 indicates that these approaches have a significant non-pharmacological effect on overall cognition in AD patients, generally targeting core impaired cognitive domains such as memory, executive function, attention, and social cognition. The intervention forms mainly include four types: cognitive training (CT), cognitive digital therapeutics, computer-aided cognitive rehabilitation (CACR), and cognitive stimulation therapy (CST). Their potential mechanisms may involve the microbiota-gut-brain axis (41), enhanced brain functional connectivity and plasticity (42), and activation-promoting nerves (43), among others. Research confirms that digital therapeutics led by computerized cognitive training, through multi-domain and adaptive training, can improve global and specific cognitive domain functions (33). CACR can use electronic devices to deliver personalized training in home and community settings and can be combined with acupuncture to achieve better results (32). Cognitive training and cognitive stimulation improve cognitive function through targeted training and group activities, respectively (32). The above evidence suggests that the four types of interventions can effectively improve patient cognition and are suitable for multiple scenarios. However, cognitive digital therapeutics currently have only expert consensus support, which is insufficient to confirm clinical efficacy; rigorous clinical trials and objective imaging indicators are still needed for further verification (44). (2) Neuromodulation technology is a core non-invasive intervention for improving cognitive function in AD patients: Evidence 5–8 shows that it is a crucial non-invasive intervention for improving global cognitive function in AD patients. It mainly includes three core forms: repetitive transcranial magnetic stimulation (rTMS), transcranial direct current stimulation (tDCS), and 40 Hz gamma transcranial alternating current stimulation (γ-tACS). All three techniques have been confirmed to effectively improve global cognitive function in AD patients. Among them, rTMS is the preferred non-invasive intervention for the dementia stage of AD and is suitable for mild-to-moderate AD patients over 60 years old. The left or bilateral dorsolateral prefrontal cortex (DLPFC) is the preferred target, typically using high-frequency stimulation to regulate neural cell electrical activity through magnetic pulses, exerting a sustained effect (45). It can precisely improve memory and brain metabolism, with effects superior to low-frequency stimulation and tDCS (28, 31, 35). Evidence 6 emphasizes that tDCS typically uses anodal stimulation on the left DLPFC to improve global cognition and verbal fluency (31), but it is not currently recommended as a standalone application for AD patients. Evidence 7 clarifies that 40 Hz γ-tACS can reduce Aβ and tau protein deposition in the brain and repair cholinergic transmission (39), making it suitable as a home-based non-invasive intervention. In clinical practice, the application of rTMS and tDCS is relatively widespread, but research on tDCS and γ-tACS regarding neuroinflammation relief remains limited, and related conclusions need further clarification (45, 46). Compared to neuromodulation, digital therapeutics offer home convenience but suffer from low evidence quality; future efforts should combine home supervision to improve compliance. rTMS has strong evidence but high costs, while tACS is suitable for home use but has weak anti-inflammatory evidence; primary care settings might consider promoting tACS combined with remote supervision.

### 4.2 Exercise and lifestyle interventions are important supports for the improvement and maintenance of cognitive function in patients with AD

(1) Exercise intervention is a fundamental measure for improving cognitive function in AD patients: Evidence 9–11 indicates that moderate-intensity aerobic exercise, strength training, Tai Chi, and Yoga can improve global cognition and delay decline in AD patients. Moderate-intensity aerobic exercise can improve global cognition and cardiopulmonary function by increasing brain-derived neurotrophic factor expression and reducing inflammatory responses. A 6-months moderate-intensity aerobic exercise regimen has the most robust evidence for cognitive improvement in mild-to-moderate AD patients. Strength training can simultaneously improve cognition, muscle disuse syndrome, balance, and mood (30). Additionally, a meta-analysis (47) confirmed that structured multimodal exercise can improve MMSE and MoCA scores in AD patients. Although it showed no significant improvement in cognitive function assessed by ADAS-Cog, it still provides high-quality evidence for global cognitive improvement in AD patients. An intervention plan of more than 150 min per week of aerobic exercise combined with resistance and balance training (2–3 times per week each), with intensity adjusted to individual conditions for at least 12 weeks, yields clear benefits. (2) Lifestyle and reminiscence interventions are important for the long-term maintenance of cognitive function in AD patients: Evidence 12–13 summarizes lifestyle and reminiscence interventions suitable for AD patients, confirming that multimodal lifestyle interventions can improve symptoms and prognosis (27) and that reminiscence interventions can effectively enhance cognition, memory, and quality of life (30). Specifically, the multimodal lifestyle intervention described in Evidence 12 includes exercise, psychological challenges, social interaction, and dietary regulation. It is easy to implement, has good compliance, and is suitable for promotion in homes and communities. Evidence 13 recommends implementing reminiscence interventions for 8–12 consecutive weeks, 1–2 times per week, with collaborative participation from family members and caregivers. However, this study did not identify literature indicating that dietary interventions improve global cognition in patients with AD; it only found that diet can play a role in AD prevention by reducing systemic inflammation and inflammatory cytokine levels and by regulating the gut microbiota (46), which weakens the evidence for lifestyle interventions. Compared with cognitive-targeted interventions, exercise and lifestyle interventions are low-cost and easy to promote in communities, but patients with severe AD require family supervision. Future research could explore a combined model of “Exercise and Lifestyle + Digital Therapeutics.”

### 4.3 Stratified collaborative intervention is an important supplement and optimization to improve cognitive function in AD patients

(1) Music and sensory stimulation are important supplements to stratified cognitive intervention for AD patients: Evidence 14–15 clarifies the forms of music and sensory stimulation interventions for AD patients and the principles of stratified implementation. Given the individual differences in cognitive function among AD patients, it is recommended to select appropriate dance, gardening, and sensory interventions based on the mild, moderate, and severe stages of the disease. Among them, dance movement therapy has promising application prospects; the multiple sensory activities involved can simultaneously activate neural pathways related to memory, emotion, and movement (48), and, through the synergistic effect of music, movement, and social interaction, improve cognition, motor function, and psychological state in mild-to-moderate AD patients. However, the specific efficacy differences among different dance forms (group dance, fitness dance, ethnic dance, ballroom dance, etc.) still need further clarification (49). Therefore, it is suggested to clarify the efficacy differences and suitable populations for different dance forms, and, based on the staging of AD, combine active and passive sensory experience forms to clarify the efficacy differences of different sensory stimulation interventions, ultimately formulating a standardized stratified intervention system covering the entire course of AD. (2) Acupuncture and comprehensive intervention are important collaborative approaches to cognitive improvement in AD patients: Evidence 16–18 summarizes acupuncture interventions and multi-non-pharmacological combined interventions for AD patients, including simple acupuncture, combined acupuncture and medicine, combined scalp and abdominal acupuncture, and multi-therapy collaborative interventions. According to existing research, acupuncture is an effective multi-target therapy for AD with few adverse reactions (50). Its mechanisms may involve modulation of microglial activity, alleviation of neuroinflammation, clearance of Aβ plaques, and neuronal protection (51), or polarization of microglia from M1 to M2 phenotype, characterized by decreased pro-inflammatory cytokines (IL-6, IL-1β, TNF-α) and increased anti-inflammatory cytokines (IL-4, IL-10) (52), thereby improving cognitive function. However, current acupuncture research is marked by methodological heterogeneity and regional cultural specificity. Some high-quality RCTs lack blinding, and efficacy is often confounded by medication factors, leaving its independent clinical benefits yet to be confirmed (50). Although combined acupuncture and medicine and multi-therapy collaboration are common forms of acupuncture treatment for AD at the primary care level and are well accepted, given drug influences, potential bias, and reproducibility, this study cautiously defines them as “collaborative optimization” rather than core recommendations. Therefore, although stratified collaborative intervention employs multimodal means, its implementation is complex. It is necessary to formulate a concise decision-making scheme based on cognitive levels to make primary care application more feasible and standardized.

## 5 Limitations

Although this study strictly followed an evidence-based design and adopted qualitative synthesis to summarize and organize existing evidence, the strength of the recommendations is limited by the quality of the included evidence. There is prominent heterogeneity in intervention protocols and outcome measures (such as significant differences in interventions among studies), limiting the feasibility of quantitative meta-analysis and precise reporting of effect sizes. In addition, although the search for study types was supplemented with free-text terms to reduce the risk of filter omissions, potential missing studies may still exist. Moreover, partial overlap of raw data across different systematic reviews and the lack of a unified definition of adverse events may pose obstacles to a complete evidence summary. Future research urgently needs to promote multi-center, high-standard RCTs to supplement and further verify the long-term effects of this protocol.

## 6 Conclusion

Based on the “5S” resource distribution model, this study summarizes 18 pieces of best evidence across 6 dimensions, constructing a complete intervention system with three levels: core intervention, basic guarantee, and collaborative optimization, providing evidence-based support for non-pharmacological interventions to improve global cognitive function in AD patients. Core intervention level: Prioritize recommending rTMS, which has the highest level of evidence, as the preferred non-invasive intervention for the dementia stage of AD. Cognitive training methods can be selected according to clinical settings (home, community, institution). tDCS should not be prescribed alone and should be used only as an adjunct. Basic guarantee level: Strongly recommend moderate-intensity aerobic exercise and strength training as basic routine interventions for mild-to-moderate AD patients. Reminiscence intervention is easy to implement and has high acceptance; it is recommended for inclusion in routine protocols for mild-to-moderate patients; for severe patients, it can be adjusted to simplified recall activities. Stratified collaborative optimization level: Mild-to-moderate AD patients can combine scalp-abdominal acupuncture protocols with multimodal interventions to enhance efficacy. Sensory stimulation interventions (music, gardening, dance, etc.) should be stratified according to the patient's cognitive level, with severe cases focusing on passive experience and sensory stimulation. Clinical practice can follow the evidence-based application logic of “baseline stratified assessment—graded prescription intervention—follow-up dynamic adjustment,” formulating individualized protocols based on the patient's disease course, care conditions, and implementation settings. Future research can further develop standardized clinical nursing pathways through Delphi expert consultation.

## Data Availability

Publicly available datasets were analyzed in this study. This data can be found here: this study did not generate any new datasets. A total of 15 eligible articles included in this review were retrieved from publicly available sources including CNKI, Wanfang Database, PubMed and UpToDate, and all references have been cited in the manuscript.
