# Supplementary material for: Non-pharmacological interventions for cognitive function improvement in patients with Alzheimer's disease: a best evidence summary
Source: Front Neurol. 2026 Sep 25;17:1888519. doi: 10.3389/fneur.2026.1888519 (PMC13649447; doi:10.3389/fneur.2026.1888519)
Supplement: Supplementary file 1 [file Table_1.docx]

**Supplementary Content**

**Supplementary Table S1 Search Strategy**

This supplementary file provides details of the search strategy executed on January 30, 2026.

| 1. CNKI | |
| --- | --- |
| #1 | AD + 阿尔茨海默病 + 阿尔茨海默症 + 老年性痴呆 + 老年失智症 |
| #2 | 非药物* + 经颅直流电刺激 + 经颅磁刺激 + 运动 + 认知干预 + 认知训练 + 数字疗法 + 饮食 + 心理社会干预 + 音乐 + 怀旧疗法 + 中医疗法 + 针灸 |
| #3 | 指南 + 专家共识 + 最佳实践 + 证据总结 + Meta + 系统评价 + 随机对照试验 |
| #4 | #1 AND #2 AND #3  75 results for search |
| Note: In the CNKI search strategy, the symbol "+" represents the Boolean operator "OR". | |
| 2. Wanfang | |
| #1 | AD or 阿尔茨海默病 or 阿尔茨海默症 or 老年性痴呆 or 老年失智症 |
| #2 | 非药物* OR 经颅直流电刺激 OR 经颅磁刺激 OR 运动 OR 认知干预 OR 认知训练 OR 数字疗法 OR 饮食 OR 心理社会干预 OR 音乐 OR 怀旧疗法 OR 中医疗法 OR 针灸 |
| #3 | 指南 or 专家共识 or 最佳实践 or 证据总结 or Meta or 系统评价 or 随机对照试验 |
| #4 | #1 AND #2 AND #3  316 results for search |
| 3. VIP | |
| #1 | AD or 阿尔茨海默病 or 阿尔茨海默症 or 老年性痴呆 or 老年失智症 |
| #2 | 非药物* OR 经颅直流电刺激 OR 经颅磁刺激 OR 运动 OR 认知干预 OR 认知训练 OR 数字疗法 OR 饮食 OR 心理社会干预 OR 音乐 OR 怀旧疗法 OR 中医疗法 OR 针灸 |
| #3 | 指南 or 专家共识 or 最佳实践 or 证据总结 or Meta or 系统评价 or 随机对照试验 |
| #4 | #1 AND #2 AND #3  57 results for search |
| 4. CBM | |
| #1 | "AD"[核心字段:智能] OR "阿尔茨海默病"[核心字段:智能] OR "阿尔茨海默症"[核心字段:智能] OR "老年性痴呆"[核心字段:智能] OR "老年失智症"[核心字段:智能] |
| #2 | "非药物*"[核心字段:智能] OR "经颅直流电刺激"[核心字段:智能] OR "经颅磁刺激"[核心字段:智能] OR "运动"[核心字段:智能] OR "认知干预"[核心字段:智能] OR "认知训练"[核心字段:智能] OR "数字疗法"[核心字段:智能] OR "饮食"[核心字段:智能] OR "心理社会干预"[核心字段:智能] OR "音乐"[核心字段:智能] OR "怀旧疗法"[核心字段:智能] OR "中医疗法"[核心字段:智能] OR "针灸"[核心字段:智能] |
| #3 | "指南"[核心字段:智能] OR "专家共识"[核心字段:智能] OR "最佳实践"[核心字段:智能] OR "证据总结"[核心字段:智能] OR "Meta"[核心字段:智能] OR "系统评价"[核心字段:智能] OR "随机对照试验"[核心字段:智能] |
| #4 | #1 AND #2 AND #3  128 results for search |
| 5. Pubmed | |
| #1 | Search: "Alzheimer Disease"[Mesh] Sort by: Most Recent |
| #2 | Search: (senile dementia[Title/Abstract]) OR (aged dementia[Title/Abstract]) |
| #3 | #1 OR #2 |
| #4 | Search: (((((((("Transcranial Direct Current Stimulation"[Mesh]))  OR "Transcranial Magnetic Stimulation"[Mesh]) OR "Exercise"[Mesh])  OR "Cognitive Training"[Mesh]) OR "Diet"[Mesh])  OR "Psychosocial Intervention"[Mesh]) OR "Music Therapy"[Mesh])  OR "Acupuncture Therapy"[Mesh] Sort by: Most Recent |
| #5 | Search: (((((non-pharmaco*[Title/Abstract]) OR (non-drug[Title/Abstract])) OR (cognitive intervention[Title/Abstract]))  OR (digital therapy[Title/Abstract]))  OR (reminiscence therapy[Title/Abstract]))  OR (traditional Chinese medicine therapy[Title/Abstract]) |
| #6 | #4 OR #5 |
| #7 | Search: ((("Randomized Controlled Trial" [Publication Type]) OR "Guideline" [Publication Type]) OR "Consensus"[Mesh]) OR  "Systematic Review" [Publication Type] Sort by: Most Recent |
| #8 | Search: ((best practice[Title/Abstract]) OR (evidence summary[Title/Abstract])) OR (Meta[Title/Abstract]) |
| #9 | #7 OR #8 |
| #10 | #3 AND #6 AND #9  560 results for search |
| 6. Embase | |
| #1 | 'alzheimer disease'/exp OR 'senile dementia'/exp |
| #2 | 'aged dementia':ab,ti |
| #3 | #1 OR #2 |
| #4 | 'transcranial direct current stimulation'/exp OR 'transcranial magnetic stimulation'/exp OR 'exercise'/exp OR 'cognitive rehabilitation'/exp OR 'diet'/exp OR 'psychosocial intervention'/exp OR 'music therapy'/exp OR 'acupuncture'/exp OR 'non pharmacological intervention'/exp OR 'non pharmacological treatment'/exp OR 'non pharmacological therapy'/exp |
| #5 | 'cognitive intervention':ab,ti OR 'digital therapy':ab,ti OR 'reminiscence therapy':ab,ti OR 'traditional chinese medicine therapy':ab,ti |
| #6 | #4 OR #5 |
| #7 | 'randomized controlled trial'/exp OR 'consensus'/exp OR 'systematic review'/exp |
| #8 | 'guideline':ab,ti OR 'best practice':ab,ti OR 'evidence summary':ab,ti OR 'meta':ab,ti |
| #9 | #7 OR #8 |
| #10 | #3 AND #6 AND #9 |
| #11 | #10 AND ('clinical trial'/lnk OR 'rehabilitation'/lnk OR 'therapy'/lnk)  587 results for search |
| 7. Cochrane Library | |
| #1 | MeSH descriptor: [Alzheimer Disease] explode all trees |
| #2 | (senile dementia):ti,ab,kw OR (aged dementia):ti,ab,kw |
| #3 | #1 OR #2 |
| #4 | MeSH descriptor: [Transcranial Direct Current Stimulation] explode all trees |
| #5 | MeSH descriptor: [Transcranial Magnetic Stimulation] explode all trees |
| #6 | MeSH descriptor: [Exercise] explode all trees |
| #7 | MeSH descriptor: [Cognitive Training] explode all trees |
| #8 | MeSH descriptor: [Diet] explode all trees |
| #9 | MeSH descriptor: [Psychosocial Intervention] explode all trees |
| #10 | MeSH descriptor: [Music Therapy] explode all trees |
| #11 | MeSH descriptor: [Acupuncture Therapy] explode all trees |
| #12 | #4 OR #5 OR #6 OR #7 OR #8 OR #9 OR #10 OR #11 |
| #13 | (non-pharmaco*):ti,ab,kw OR (non-drug):ti,ab,kw OR (cognitive intervention):ti,ab,kw OR (digital therapy):ti,ab,kw OR (reminiscence therapy):ti,ab,kw OR (traditional Chinese medicine therapy):ti,ab,kw |
| #14 | #12 OR #13 |
| #15 | MeSH descriptor: [Randomized Controlled Trial] explode all trees |
| #16 | MeSH descriptor: [Guideline] explode all trees |
| #17 | MeSH descriptor: [Consensus] explode all trees |
| #18 | MeSH descriptor: [Systematic Review] explode all trees |
| #19 | #15 OR #16 OR #17 OR #18 |
| #20 | (best practice):ti,ab,kw OR (evidence summary):ti,ab,kw OR (Meta):ti,ab,kw |
| #21 | #19 OR #20 |
| #22 | #3 AND #14 AND #21  157 results for search |
| 8. CINAHL | |
| S1 | (MH "Alzheimer's Disease") |
| S2 | TI (senile dementia OR aged dementia) OR AB (senile dementia OR aged dementia) |
| S3 | S1 OR S2 |
| S4 | (MH "Transcranial Direct Current Stimulation") OR (MH "Transcranial Magnetic Stimulation") OR (MH "Exercise") OR (MH "Cognitive Therapy") OR (MH "Diet") OR (MH "Psychosocial Intervention") OR (MH "Music Therapy") OR (MH "Acupuncture") |
| S5 | TI (non-pharmaco* OR non-drug OR cognitive intervention OR digital therapy OR reminiscence therapy OR traditional Chinese medicine therapy) OR AB (non-pharmaco* OR non-drug OR cognitive intervention OR digital therapy OR reminiscence therapy OR traditional Chinese medicine therapy) |
| S6 | S4 OR S5 |
| S7 | (MH "Randomized Controlled Trials") OR (MH "Consensus") OR (MH "Systematic Review") |
| S8 | TI (Guideline OR best practice OR evidence summary OR Meta) OR AB (Guideline OR best practice OR evidence summary OR Meta) |
| S9 | S7 OR S8 |
| S10 | S3 AND S6 AND S9 |
| S11 | S10 AND (full text)  133 results for search |
| 9. web of science | |
| #1 | ((TS=("Alzheimer Disease")) OR TS=("senile dementia")) OR TS=("aged dementia") |
| #2 | (((((((((((((TS=("Transcranial Direct Current Stimulation"))  OR TS=("Transcranial Magnetic Stimulation")) OR TS=(Exercise))  OR TS=("Cognitive Training")) OR TS=(Diet))  OR TS=("Psychosocial Intervention")) OR TS=("Music Therapy"))  OR TS=("Acupuncture Therapy")) OR TS=(non-pharmaco*))  OR TS=(non-drug)) OR TS=("cognitive intervention"))  OR TS=("digital therapy")) OR TS=("reminiscence therapy"))  OR TS=("traditional Chinese medicine therapy") |
| #3 | ((((((TS=("Randomized Controlled Trial")) OR TS=(Guideline)) OR TS=(Consensus)) OR TS=("Systematic Review")) OR TS=("best practice")) OR TS=("evidence summary")) OR TS=(Meta) |
| #4 | #1 AND #2 AND #3  192 results for search |
| 10. JBI | |
| #1 | Alzheimer Disease.sh. |
| #2 | (Transcranial Direct Current Stimulation or Transcranial Magnetic Stimulation or Exercise or Cognitive Training or Diet or Psychosocial Intervention or Music Therapy or Acupuncture Therapy).sh. |
| #3 | (non-pharmaco$ or non-drug or cognitive intervention or digital therapy or reminiscence therapy or traditional Chinese medicine therapy).ab,kw,ti. |
| #4 | #2 OR #3 |
| #5 | #1 AND #4  2 results for search |
| 11. ADI | 1 results for search |
| 12. BMJ | 5 results for search |
| 13. NICE | 3 results for search |
| 14. GIN | 6 results for search |
| 15. RNAO | 1 results for search |
| 16. Up to Date | 4 results for search |
| 17. Medlive | 8 results for search |
| Total | 2235 results for search |
