# Supplementary material for: Non-pharmacological interventions for cognitive function improvement in patients with Alzheimer's disease: a best evidence summary
Source: Front Neurol. 2026 Sep 25;17:1888519. doi: 10.3389/fneur.2026.1888519 (PMC13649447; doi:10.3389/fneur.2026.1888519)
Supplement: Supplementary file 2 [file Table_2.docx]

**Supplementary Table S2 JBI Evidence FAME Structure (JBI FAME Scale)**

| **Evidence FAME Structure** | **Description** |
| --- | --- |
| Feasibility of Evidence  F-Feasibility | What is the cost-effectiveness of implementing this practice?  Are the resources required to implement this practice accessible?  Is there sufficient experience and capability to implement this practice? |
| Appropriateness of Evidence  A-Appropriateness | Is this practice culturally acceptable?  Can this practice be translated or applied to the majority of the population?  Is this practice suitable for various settings? |
| Clinical Meaningfulness of Evidence  M-Meaningfulness | Is the application of this practice associated with positive patient experiences?  Will the application of this practice avoid adverse patient experiences? |
| Effectiveness of Evidence  E-Effectiveness | Is there a benefit to using this practice?  Is this practice safe? |
