# Supplementary material for: Non-pharmacological interventions for cognitive function improvement in patients with Alzheimer's disease: a best evidence summary
Source: Front Neurol. 2026 Sep 25;17:1888519. doi: 10.3389/fneur.2026.1888519 (PMC13649447; doi:10.3389/fneur.2026.1888519)
Supplement: Supplementary file 3 [file Table_3.docx]

**Supplementary Table S3 Evidence Recommendation Levels of JBI 2014 Edition**

| **Recommendation Level** | **Judgment Criteria** |
| --- | --- |
| Grade A recommendation:  Strong recommendation | 1. Clearly demonstrate that the intervention measures are more beneficial than harmful, or more harmful than beneficial.  2. High-quality evidence supports the application.  3. It is beneficial or has no impact on resource allocation.  4. The patient's values, wishes, and experiences were taken into consideration. |
| Grade B recommendation:  Weak recommendation | 1. Intervention measures have more advantages than disadvantages, or more disadvantages than advantages, although the evidence is not yet clear.  2. There is evidence supporting the application, although the quality of the evidence is not high.  3. It is beneficial for resource allocation, or has no impact, or has a minor impact.  4. Partially considers, or does not consider, the patient's values, wishes, and experiences. |
