# Supplementary material for: Non-pharmacological interventions for cognitive function improvement in patients with Alzheimer's disease: a best evidence summary
Source: Front Neurol. 2026 Sep 25;17:1888519. doi: 10.3389/fneur.2026.1888519 (PMC13649447; doi:10.3389/fneur.2026.1888519)
Supplement: Supplementary file 4 [file Table_4.docx]

**Supplementary Table S4 JBI FAME Evidence Evaluation Results on the Impact of Non-pharmacological Interventions on Cognitive Function in Patients with Alzheimer's Disease**

| **Evidence item No.** | **Evidence subject** | **F** | **A** | **M** | **E** |
| --- | --- | --- | --- | --- | --- |
| 1 | Cognitive Training (CT) | High | High | High | High |
| 2 | Cognitive Digital Therapy | Medium | High | High | Low |
| 3 | Computer-Assisted Cognitive Rehabilitation (CACR) | High | High | High | High |
| 4 | Cognitive Stimulation (CST) | High | High | High | High |
| 5 | rTMS | High | High | High | High |
| 6 | tDCS | Medium | High | High | Medium |
| 7 | 𝛾-tACS (40Hz) | Medium | Low | High | High |
| 8 | Comprehensive evaluation of rTMS/tDCS/𝛾-tACS efficacy | High | High | High | High |
| 9 | Moderate-intensity aerobic exercise | High | High | High | High |
| 10 | Moderate-intensity strength training | High | High | High | High |
| 11 | Exercise prescription | High | High | High | High |
| 12 | Lifestyle intervention(diet/cognitive challenge, etc.) | High | Medium | High | High |
| 13 | Reminiscence and memory guidance | High | High | High | High |
| 14 | Dance therapy | Medium | High | High | Medium |
| 15 | Horticultural Therapy | High | High | High | High |
| 16 | Simple Acupuncture | High | High | High | Medium |
| 17 | Combined Intervention(music/reminiscence/neuromodulation) | High | High | High | High |
| 18 | Combined Scalp and Abdominal Acupuncture | High | High | High | High |
